# Tracking iron oxide labelled mesenchymal stem cells(MSCs) using magnetic resonance imaging (MRI) in a rat model of hepatic cirrhosis

**DOI:** 10.6026/97320630015001

**Published:** 2019-01-31

**Authors:** Abdulwahab Noorwali, Mamdooh Faidah, Naushad Ahmed, Abdulhadi Bima

**Affiliations:** 1 Stem Cell Unit, King Fahd Medical Research Centre, King Abdulaziz University, Jeddah 21589, Saudi Arabia; 2 Department of Medical Laboratory,College of Health Sciences,King Abdulaziz University,Jeddah 21589 Saudi Arabia; 3 Department of Radiology,King Abdulaziz University Hospital,King Abdulaziz University,Jeddah 21589, Saudi Arabia; 4 Department of Clinical Biochemistry,King Abdulaziz University Hospital,King Abdulaziz University,Jeddah 21 89,Saudi Arabia

**Keywords:** Stem cells, Liver Cirrhosis, Nanoparticles, Magnetic Resonance Imaging, Transmission Electron Microscopy, in vivo

## Abstract

Homing and tumor attenuation potential of BM-MSCs labelled with superparamagnetic iron-oxide nanoparticles (SPIONs) in a rat model of hepatic cirrhosis was evaluated. Rat BM-MSCs were derived, characterized and labelled with SPIONs (200 nm; 25 mg Fe/ml). Hepatic cirrhosis was induced in Wistar rats (n=30; 10/group) with carbon tetrachloride (CCl4; 0.3 mL/kg body weight) injected twice a week for 12 weeks. Group-I was administered vehicle (castor-oil) alone; Group-II received two doses of unlabelled BM-MSCs (3x106 cells) and Group-III received two doses of SPIONs labelled BM-MSCs (3x106 cells) via tail vein injection (0.5 ml) at weekly intervals. All animals were sacrificed after two weeks for histological, radiological and biochemical analysis. Derived BM-MSCs demonstrated MSCs related CD markers. Histology confirmed induction of hepatic cirrhosis with CCL4. Levels of alanine-aminotransferase, aspartate-aminotransferase,alkaline-phosphatase and gamma glutamyl-transferase returned to normal levels following treatment with BM-MSCs. Uptake and homing of SPIONs labelled BM-MSCs, and reduction in the size of cirrhotic nodules were confirmed using transmission electron microscopy and magnetic resonance imaging respectively. BM-MSCs reduced the pathological effects of CCL4 induced hepatic cirrhosis and labelling BMMSCs with SPIONs were non-toxic and enabled efficient tracking using non-invasive methods.

## Background

Liver is one of the most important organs in the human body, which helps removal of harmful/toxic substances, and thereby maintains homeostasis. Liver is involved in various functions such as synthesis of bile, cholesterol, conversion of glucose into glycogen and its storage within its parenchyma as well as breakdown and recycle of aged/damaged red blood cells. Given its multifaceted functions it is quite natural for the liver to be exposed to both microbial and toxic substances, which can lead to altered liver structure and function. Intake of alcohol, drugs, bacterial or viral infections can thus lead to liver injury and/or toxic hepatopathy [1]. When such injury becomes persistent or chronic it leads to liver fibrosis/cirrhosis and in later stages can also transform into benign or malignant cancer and therefore is a major clinical concern.

Current therapies including both allopathic and natural remedies help restoration of the liver function by taking advantage of its inherent regeneration potential. However, the reversal of lost architecture is minimal to absent when the liver damage is in its advanced stages of fibrosis/cirrhosis leading on to liver cancer. Administration of either differentiated or undifferentiated stem cells to alleviate the underlying pathology is largely being explored [2]. Stem cells and/or their derivatives can be modified to act as pro-apoptotic, anti-angiogenic and anti-proliferative agents against different cancers. As such cellular fractions and/or drug loaded nanoparticles or scaffolds are used as therapeutic agents or drug carriers [3-6].

Direct local injection of stem cells or other therapeutic agents is always not possible. In many instances, the stem cells are administered via the common accessible routes of drug administration, either as intravenous, intraperitoneal or intraarticular injections. Stem cells have inherent migratory properties toward tumor cells [7]. In addition, the cross-talk between stromal derived factor-1 (SDF-1) and cytokines/chemokines help in migration of stem cells to the afflicted sites [8]. Although stem cells per se have inherent migratory properties toward tumor cells, in most cases it remains to be verified whether the stem cells reach their intended site of action. Tracking of these stem cells/cellular products using advanced labelling materials and techniques therefore becomes essential.

Of the various advanced imaging techniques with high safety profile, positron emission tomography (PET) and MRI are mostly preferred for in vivo imaging studies [9]. In comparison to PET, MRI is the most popular and is used in conjunction with magnetic labelling, and is a powerful technique for non-invasive tracking of cells in vivo [10, 11]. Magnetic nanoparticles have been used for both diagnosis and treatment with promising results [12-15]. Most commonly used are the ferrite and iron oxide nanoparticles, which due to their superparamagnetic properties are utilized for diagnostic and therapeutic uses. Moreover, these nanoparticles have low toxicity, larger surface area to volume ratio, less sensitivity to oxidation and therefore superparamagnetic iron oxide nanoparticles (SPIONs) are promising candidates for use in biomedical applications including molecular imaging [16-19].

Although MSCs continue to be used for their therapeutic benefits against various diseases, it remains to be ascertained whether the delivered MSCs reach their intended site of action. As such in the present study we explored the therapeutic role of MSCs labelled with SPIONs in the amelioration of hepatic injury/cirrhosis in rats, through MRI tracking of cells labelled with iron oxide nanoparticles (SPIONs). The combination of cell labelling and MRI tracking in a time dependent manner with close follow up of the lesion size provide a novel, more objective assessment of the role of MSCs in disease control.

## Methodology

### Animals:

All animal experiments were conducted in accordance with the regulations of the international animal use and care committee and approved by the Institutional Research Ethics Committee, King Abdulaziz University (KAU), Jeddah, Saudi Arabia vide protocol number HA-02-J-008. Thirty female Wistar rats, 14 to 16 weeks that weighed between 300 to 400 grams were used in the study. All rats were housed in the animal holding unit of King Fahd Medical Research Center (KFMRC), KAU, under a 12 h light/dark cycle at a temperature of 25°C and relative humidity ranging from 60% to 70% throughout the experiment. The animals had free access to standard rat pellet and water ad libitum.

### Culture of bone marrow mesenchymal stem cells (BM-MSCs):

BM-MSCs derived from the bone marrow washings of femur and tibia of 6-8 weeks old Wistar rats were used for iron oxide labelling and their tracking in vivo. The BM-MSCs were thawed and expanded in culture using Dulbecco's modified Eagle's media (DMEM) containing 10% fetal bovine serum (FBS), 2mM Lglutamine and antibiotics [penicillin (50IU/streptomycin (50µg/ml)].

### CD marker analysis using fluorescent activated cell sorting:

Early passage (P2) of the derived BM-MSCs was analysed for CD marker expression. Briefly, the cells in culture were trypsinized, aliquoted into different tubes (1x105 cells/tube) in 3% FBS and labelled with the antibodies (10 µl each) namely, CD29, CD44, CD73, CD34 and CD45 (BD Biosciences, San Jose, CA). Following incubation with respective antibodies for 20 min at 4°C in the dark, the cells were centrifuged (1000 rpm x 5 min). The cells were then resuspended in 3% FBS before analysis using FACSAria III flow cytometer and FACSDIVA software (BD Biosciences, San Jose, CA).

### Differentiation into adipocytes, chondrocytes and osteocytes:

The BM-MSCs (2x10^4^ cells) from early passages (P3-P4) were plated into 24-well plates and cultured using respective differentiation media. Adipogeneic media consisted Dulbecco's modified essential medium with low glucose (DMEM-LG), dexamethasone (1 µM), indomethacin (0.2 mM), insulin (10 µg/ml), 3-isobutyl-1- methylxanthine (0.5 mM) (Cambrex Biosciences, Baltimore, MD); chondrogeneic media comprised of DMEM-LG, the Insulin Transferrin-Selenium premix and TGF-β (10 ng/ml) (Peprotech, Rocky Hill, NJ);and the osteogeneic media contained DMEM-LG, ascorbic acid (50 mM), dexamethasone (100 nM) and β- glycerophosphate (10 mM). The cells were cultured for 3 weeks and stained using Oil Red O, Alcian Blue, or Alizarin red for adipogeneic, chondrogenic or osteogenic differentiation respectively and imaged using microscope.

### Labelling of MSCs with iron oxide nanoparticles:

Iron oxide nano particles [Fluid MAG-nano particles (Chemicell, Berlin, Germany)] was used to label the BM-MSCs. Briefly, the BMMSCs were counted and seeded at a density of 1x10^5^ cells/well in a 24-well plate and incubated overnight in a CO_2_ incubator under standard culture conditions of 37°C and 5%CO_2_ in atmospheric air. Following fresh changes of medium, iron oxide nanoparticles (SPIONs) were added at a concentration of 25 µg Fe/ml. In addition, 0.75 mg/ml Poly-l-lysine was added and the cells cultured for 24 h. Fluid MAG-nano particles (Chemicell, Berlin, Germany) are ferrofluids, 200-nm-diameter particles consisting of an aqueous dispersion of magnetic iron oxides coated by hydrophilic polymers which protect them against aggregation by foreign ions [20]. Poly-l-lysine was utilized as a prospective vehicle for SPIONs transport into cells, and enable endocytosis of iron oxide nanoparticles by MSCs [21].

### Cell viability:

BM-MSCs were seeded (1x10^4^ per well) in a 96-well tissue culture plate and cultured for 24 h, 48 h and 72 h under standard culture conditions of 37°C and 5% CO_2_ in atmospheric air. Cell proliferation of both unlabelled and SPION labelled BM-MSCs was analysed by Micro-culture Tetrazolium Assay (MTT, Sigma Chemical Company, St Louis, MO, USA). Briefly, 10 µl MTT reagent (5mg/ml) was added to cells in fresh media (100 µl) and cultured for 4 h. The formazan crystals formed were then solubilized with DMSO and the optical density determined at 570 nm with a reference wavelength of 630 nm using SpectraMaxi3 Multimode Reader (Molecular Devices, USA).

### Transmission Electron microscopy (TEM):

Briefly, both unlabelled and labelled BM-MSCs in culture were trypsinized, washed thrice with PBS, centrifuged, fixed in 2.5% glutaraldehyde and post-fixed in 2% osmium tetroxide. The samples were then dehydrated in graded series of ethanol and embedded in epoxy resin. Ultrathin (50 -70 nm) sections collected on metal mesh grid was stained and imaged using Philips CM100 TEM (Holland).

### Induction of hepatic cirrhosis:

Hepatic cirrhosis was induced in all rats by subcutaneous injection of 0.3 mL/kg body weight of carbon tetrachloride (CCl_4_) in castor oil using previously established protocol [22]. CCl_4_ was administered thrice in the first week and thereafter twice weekly for 12 weeks. Following induction of hepatic injury, the rats were randomly divided into three different experimental groups of ten each. Group-I was administered vehicle alone and served as control; Group-II received two doses of unlabelled BM-MSCs (3x10^6^ cells) and Group-III received two doses of SPIONs labelled BMMSCs (3x10^6^ cells) via tail vein injections (0.5 ml, PBS). The cells were injected once a week and each rat therefore received two cell administrations in total.

### Liver enzymes:

The serum samples obtained from the control and CCl_4_ treated rats were used in the biochemical assays. Alanine aminotransferase (ALT, K2143, Siemens) and aspartate aminotransferase (AST, K2041, Siemens) activities were assayed according to earlier published method [23]. Gamaglutamyl transpeptidase (GGT, K2045, Siemens) and alkaline phosphatase (ALP, K2115, Siemens) activities were determined using kinetic methods respectively [24, 25]. Absorbance was determined spectrophotometrically using SpectraMaxi3 Multimode Reader (Molecular Devices, USA).

### Liver histology:

Formalin fixed liver tissue from normal control, CCL_4_ and BMMSCs treated groups were dehydrated in ethanol series, washed in xylene and embedded in paraffin wax. Tissue blocks were sectioned at 5-6 µM thickness, deparaffinized and stained with hematoxylin and eosin and analyzed under microscope (Olympus Instruments, Tokyo, Japan).

### Quantitative Real Time-PCR (qRT-PCR):

Total RNA was extracted from the liver tissue of both control and MSCs treated rats using MagNA Pure compact RNA isolation kit (Roche, Penzberg, Germany) according to the manufacturer's protocol. First-strand cDNA synthesis was carried out using High- Capacity cDNA Reverse Transcription Kit (Applied Biosystems, Foster City, CA). Primers were designed using previously published primers, and the primer sequences are given in Table 1. The qRT-PCR analysis was performed using the ABI StepOnePlusTM Real-Time PCR System (Applied Biosystems, Foster City, CA) using TaqMan® Universal PCR Master Mixand relative quantification was performed using the comparative 2^-ΔΔ^Ct method.

### Magnetic Resonance Imaging (MRI):

MRI was used to track both SPIONs labelled and unlabelled BMMSCs in a time dependent manner after tail vein injection. The scan was performed using Biospec 94/30 USR research scanner with T2 Turbo rapid acquisition with relaxation enhancement (RARE) sequences. Axial T2 Turbo RARE images were obtained on day 0, 2 and 6 days after BM-SCs injection. In addition, the scans were performed at 30, 60 and 90 min following nano-labelled BM-MSCs injection for the rats in Group-III. Care was taken to ensure that the sequence parameters were standardized for all scans (TR - 110 ms, TE - 27 ms, FA - 180 degrees, NEX 4) [26, 27]. Post processing software was used to analyze the images. Appropriate slice matched images were chosen in each of the scans and the signal intensity was calculated using a regions of interest (ROI) tool.

### Statistical analysis:

The social package for statistical sciences (SPSS, version 16) was used to compute statistics. All values were presented as Mean ± standard error of the mean (SEM) from three different experimental samples. Asterisk (*) indicates statistical significance of p<0.05.

## Results

### BM-MSCs culture, characterization and differentiation:

**Figure 1:**
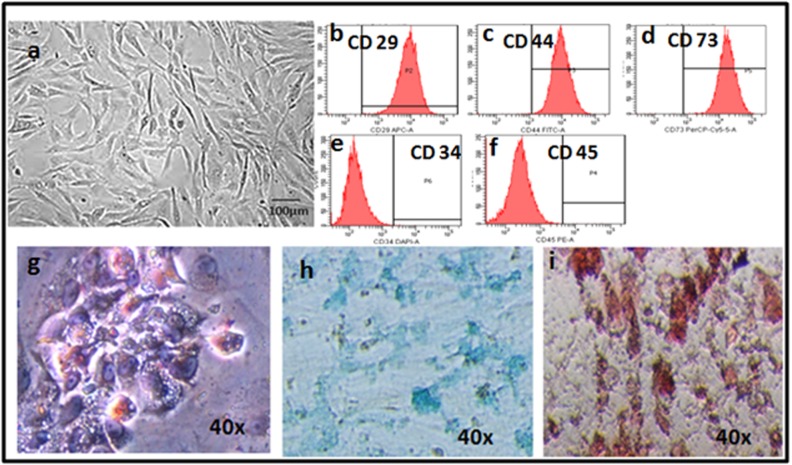
(a)Phase contrast image of rat bone marrow mesenchymal stem cells (BM-MSCs) showing the characteristic spindle shaped short fibroblasts; (b-f) fluorescent activated cell sorting (FACS) images showing both the positive (CD29, CD44, CD73) and negative (CD34, CD45) MSC related CD makers; (g-i)histological images of BM-MSCs differentiated into adipocytes (g), chondrocytes (h) and osteocytes (i) and stained with oil Red O, alcian blue and alizarin red respectively (magnification 40x).

Bone marrow derived MSCs (BM-MSCs) demonstrated their characteristic spindle shaped morphology (Figure 1a) and were plastic adherent. The BM-MSCs were positive for the CD makers CD29, CD44, CD73 and negative for CD34 and CD45 (Figure 1b-f), and could be differentiated into adipocytes, chondrocytes and osteocytes (Figure 1g-i). MSCs cultured in adipogenic media showed changes in morphology starting from 8-10 days and by the end of second and third week almost 90% of the cells demonstrated the characteristic vacuolations upon staining with Oil Red O (Figure 1g). Cells cultured in chondrogenic media lost their spindle shape and became polygonal and these cells demonstrated positive staining with Alcian blue which is indicative of collagen matrix deposition (Figure 1h). The MSCs cultured in osteogenic media showed dense deposits in culture which stained positive with Alizarin red indicative of calcium deposition (Figure 1i).

### BM-MSCs labelling with SPIONs and TEM:

**Figure 2:**
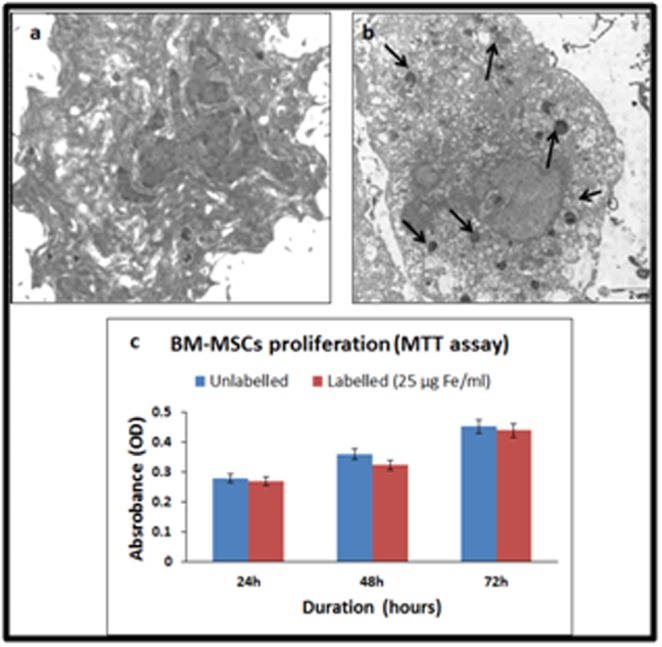
Transmission electron microscopic images of (a) unlabelled and (b) super paramagnetic iron oxide nano particles (SPIONs) labelled BM-MSCs. Arrows indicate the internalized SPIONs; (c) MTT assay of both unlabelled and SPIONs labelled BM-MSCs at 24 h, 48 h and 72 h showing increase in cell proliferation with time.

The SPIONs were readily taken up by the BM-MSCs and their cellular localizations were demonstrated by TEM. The SPIONs labelled BM-MSCs showed even distribution of the internalized nanoparticles in the cytoplasm which appeared as dark dense spherical bodies (Figure 2a, b).

### BM-MSCs proliferation assay following labelling with SPIONs:

BM-MSCs cultured with SPIONs demonstrated normal cell proliferation like that of the control. No changes in morphology or any adverse effects leading to cell death were observed indicating that iron oxide cell labelling was effective without any compromise on cellular viability (Figure 2c).

### Liver enzymes:

**Figure 3:**
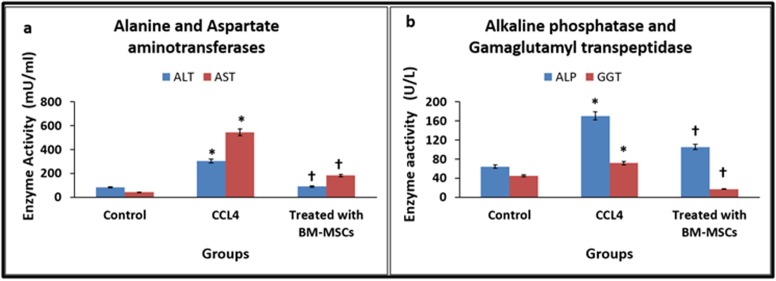
(a) Serum levels of alanine amino transferase and aspartate amino transferase following treatment with bone marrowmesenchymal stem cells in control and carbon tetrachloride (CCl4) induced cirrhosis in rats; (b) Serum levels of alkaline phosphatase and gamaglutamyl transpeptidase following treatment with bone marrow-mesenchymal stem cells in control and carbon tetrachloride induced cirrhosis in rats.

The levels of ALT and AST were increased in CCl4 treated group compared to the normal control. However, treatment with BMMSCs led to decrease in both ALT and AST to near normal levels. The values of ALT were 82.0, 304.25, 89.25 mU/mL and AST were 41.25, 545.50, 182.0 mU/mL, for normal control, CCl_4_ treated group and BM-MSCs treated group respectively. There was a mean percentage increase in CCl4 treated group by 371.04% for ALT and 1322.42% for AST compared to normal control and these increases in values were statistically significant (Figure 3a). However, compared to the CCl_4_ treated group there was a mean percentage decrease by 70.67% for ALT and 66.64% for AST and these decreases were statistically significant (Figure 3a).

The levels of ALP and GGT demonstrated an increase in CCl4 treated group compared to the normal control. However, treatment with BM-MSCs led to decrease in both ALP and GGT to near normal levels. The values of ALP were 64.25, 170.25, 105.50 U/Land GGT were 45.0, 71.75, 17.25 U/L, for normal control, CCl_4_ treated group and BM-MSCs treated group respectively. There was a mean percentage increase in CCl_4_ treated group by 264.98% for ALP and 159.44% for GGT compared to normal control and these increase in values were statistically significant (Figure 3b). However, compared to the CCl_4_ treated group there was a mean percentage decrease by 38.03% for ALP and 75.96% for GGT and these changes were statistically significant (Figure 3b).

### Liver injury/cirrhosis:

**Figure 4:**
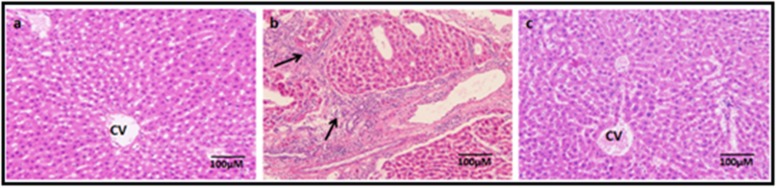
Haematoxylin and eosin stained liver sections of normal (a), fibrotic liver following carbon tetrachloride (CCl4) injections (b) and regenerated liver following treatment with BM-MSCs (c). Dense areas of fibrosis (indicated by arrows) and disorganized liver pattern was observed following CCl4 induced liver injury (b), while normal hepatic architecture with radial arrangement of hepatocytes as that of normal liver was seen following liver regeneration with BM-MSCs treatment (c). Magnification 10x.

The liver samples from untreated controls showed normal histological pattern of liver lobules with radially arranged hepatocytes, the central portal vein and peripheral portal triad (Figure 4a). In contrast, the CCl_4_ treated group the liver lobules showed more trabecular pattern with loss of hepatocytes and more connective tissue infiltration (Figure 4b). However, the group treated with MSCs following CCl_4_ induced liver injury, showed reversal towards normal liver pattern (Figure 4c).

### Magnetic Resonance imaging (MRI):

**Figure 5:**
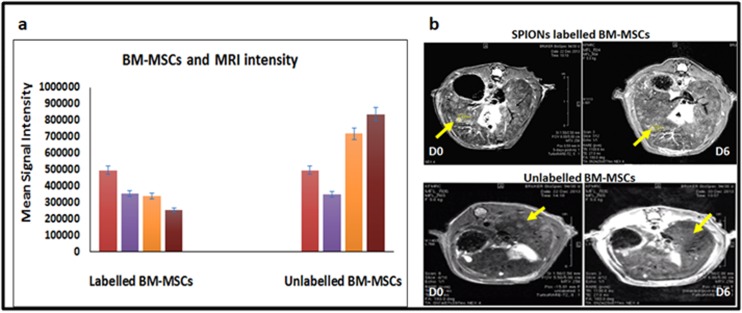
ChartofMean signal Intensity of time course MR imaging demonstrating the homing of labelled and unlabelled BM-MSCs to the injured liver (a); Time course MRI performed on day 0 and day 6 after 2nd dose SPIONs labelled and unlabeled BM-MSCs showing the Axial T2 Turbo RARE images. Arrows indicate decreases in size of the nodules with varying intensity in labelled in unlabeled BM-MSCs respectively (b).

MRI done following administration of iron oxide labelled BMMSCs demonstrated a sequential fall in liver intensity, compared to the unlabelled MSCs. In the unlabelled rats the signal intensities of the liver remained increased over the 6-day period of scanning (Figure 5a). In labelled rats, there was a sequential fall in the signal intensity of the liver (Figure 5a). Axial T2 Turbo RARE images done on rats in CCl_4_ treated groups demonstrated multiple dysplastic nodules in both lobes of a cirrhotic liver in the BM-MSCs preinjection scan (Day 0). Following administration of both unlabelled and labelled BM-MSCs, the size of these nodules decreased with time from 2.63 mm (day 0) to 1.39 mm (day 6) (Figure 5C). Sequential fall in the signal intensity of the liver nodules was noted in rats that received SPIONs labelled BM-MSCs, while the signal intensity was increased in rats that received unlabelled BM-MSCs (Figure 5b).

### Quantitative real-time PCR:

**Figure 6:**
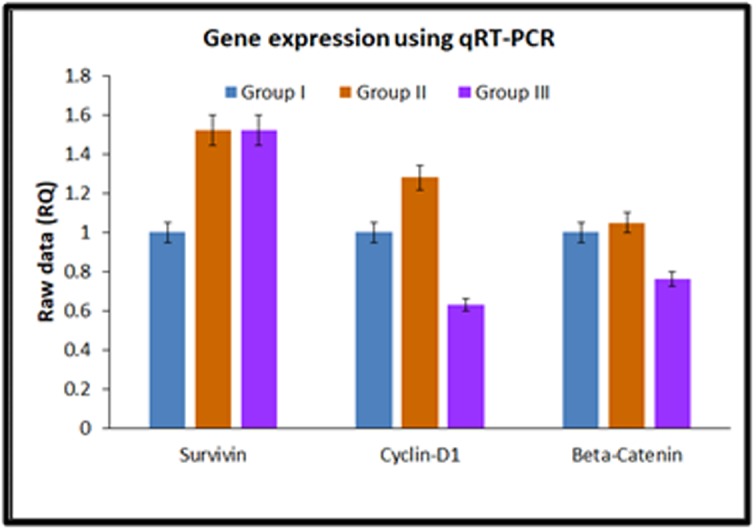
Gene expression analysis showing the expression of survivin, cyclinD1 and beta Catenin in control (Group-I), unlabelled BM-MSCs (Group-II) and SPIONs labelled BM-MSCs (Group-III).

Real time gene expression analysis showed an increase in survivin and a decrease in β-catenin gene expression, while cyclin-D1 showed a biphasic change compared to the control. Survivin was increased by 1.5 fold in both Group-II and Group-III. Cyclin-D1 was increased by 1.3 fold and decreased by 0.63 fold in Group-II and Group-III respectively. β-catenin showed no changes in Group- II compared to control while it was decreased by 0.63 fold in Group-III. The observed differential expression of genes were however not statistically significant (Figure 6).

## Discussion

Chronic liver injury due to viral infections, alcohol intake or toxic agents can cause liver failure, cirrhosis and/or eventually lead to hepatocellular carcinoma [28]. Liver in general has good inherent regenerative capacity and the presence of hepatic progenitor cells helps restoration of liver structure and function [29, 30]. However, this self-regeneration is true only in the early stages of liver disease, whereas in advanced stages the liver structure and function are maximally compromised and self-regeneration is poor [31]. Under such circumstances, the use of autologus/allogeneic stem cells or their derivatives may help liver regeneration [2, 32]. However, it is imperative to understand whether transplanted/delivered stem cells effectively reach their intended targets, integrate with the host tissue and contribute to the restoration of function. Towards this end, much recent advancement in cell tracking has been achieved. In the present study, we utilized MSCs to alleviate cirrhosis in a rat model and we have successfully tracked the homing of the iron oxide (SPIONs) labelled MSCs using MRI. We also observed that homed MSCs helped reduction of the induced cirrhotic lesions as well as contributed to liver regeneration.

In the present study, the iron oxide nano particles cultured with BM-MSCs were readily internalized. This was facilitated by the presence of poly-L-lysine, which is well known to stimulate the cellular endocytosis of iron-oxide nano particles [21]. Furthermore, the iron oxide nano particles were inert and the BM-MSCs exhibited normal cell viability and proliferation, as reported earlier [33].

Although molecular imaging techniques have evolved significantly during the last decade, no single imaging modality can provide all the information required to track transplanted stem cells and monitor their functional effects. Each imaging modality used for stem cell tracing has its advantages and disadvantages [34,35]. In the present study, the MRI Axial T2 Turbo RARE images obtained following injection of SPIONs labelled and unlabelled BM-MSCs via the rat tail vein on day 0, 2 and 6 showed an initial decline in signal intensity up to 48 hours followed by an increase in intensity thereafter until the end of the end of study period. The decline in signal intensity could be explained by the homing of iron-labelled cells into the liver in the first 24-48 hours. Administration of MSCs labelled with SPIONs into the portal vein and renal artery are reported to exhibit hypo intensity of liver with T2 weighted MRI images [36]. The turbo RARE images helped to achieve the nanoparticles signals effectively, enabling assessment of the diminishing size of the cirrhotic nodules and evaluation of regenerating liver nodules in the present study. The paramagnetic properties cause differences in homogeneities which leads to hypo intensity in T2 weighted images, and the effectiveness of the procedure depends on numerous factors including the size, dose, charge and coating of the nanoparticles [37]. The increase in intensity thereafter may be explained by the improvement in liver pathology associated with MSCs injection as well as the clearance of iron from the injected cells, possibly through extrusion and biliary excretion or engulfment by hepatic Kupffer cells [36].

Measuring the size of a representative focal lesion in the liver and the progressive reduction in its diameter associated with stem cell therapy in the present study, confirms and correlates with the amelioration of hepatic pathology [38]. In addition, the elevated liver enzymes (ALP, ALT, AST and GGT) associated with induction of cirrhosis were restored to normal levels following BM-MSCs therapy, indicating improvement in liver function. The gene expression however, did not show much correlation with the biochemical or the histological pattern observed. Survivin gene expression was increased in rats treated with both labelled and unlabelled BM-MSCs, indicating that the tumor remained active despite treatment with stem cells.Our results were in contrast to an earlier study on a mice model, which demonstrated decreased survivin expression following treatment with MSCs [39]. The dose of administered MSCs and the stage of the tumour may probably play a role in survivin expression. Survivin, which is an inhibitor of apoptosis is normally highly expressed during tumor development and progression [40] and interestingly, the small molecule YM155 is identified to be effective against HCC with over expression of survivin [41], thus opening new therapeutic strategies.

Cyclin D1 and β-catenin showed decreased expression following treatment with labelled BM-MSCs compared to control. Cyclin D1 is usually over expressed in advanced cases of HCC and is associated increased cell proliferation and poor clinical outcome [42]. β-catenin, plays a key role in Wnt signalling pathway and its over expression is associated with cancer initiation, progression and cancer stem cell maintenance, including HCC [43, 44]. MSCs over expressing hepatocyte nuclear factor 4α, is demonstrated to inhibit HCC by downregulation of β-catenin, cyclin D1 and matrix metalloproteinases [45]. The mechanisms by which BM-MSCs brought about HCC inhibition in the present study and whether the decreased cyclin D1 and β-catenin expression is a direct effect of stem cell treatment or a result of other aberrant gene activation needs further studies.

In general, MSCs have known to exert their benefits either by undergoing differentiation in the host tissue or by a paracrine mechanism by release of cytokines/chemokines that help reduce the inflammation, fibrosis and oxidative stress associated with the disease [28]. Furthermore, MSCs are hypoimmunogenic in nature and both autologous and allogeneic MSCs from various sources have been used in the past for the treatment of liver related diseases with variable success [28, 46, 47]. More ongoing clinical trials indicate that stem cells will find increasing applications in liver diseases and therefore use of imaging techniqueswill allow effective monitoring of the administered stem cells and progress in disease management.

## Conclusion

Cell based therapies for liver diseases are rapidly increasing and some studies have progressed to Phase I clinical trials. Our study highlights the use of BM-MSCs in the amelioration of liver cirrhosis and how the stem cells can be labelled with SPIONs and tracked using MRI. The T2 weighted MRI sequences are best suited to evaluate liver cirrhosis/liver tumours and SPION labelled MSCs can be tracked in real time to confirm homing of transplanted cells and follow up with disease prognosis.

## Conflict of Interest

All authors have no conflict of interest

## Author contributions:

All authors contributed equally to the conceptualization, design, and coordination of this study. MF, NA, MAQ performed the experiments, analysed the data and prepared manuscript draft. AN, GK supervised the study, provided intellectual support, reviewed and edited the manuscript. AM provided intellectual support; reviewed and edited the manuscript.

## Figures and Tables

**Table 1:** Gene names and primer sequences used for quantitative real time PCR. F: Forward primer; R: Reverse primer

Genes	Primer sequences
Survivin	F: 5'-GAGCAGCTGGCTGCCTTA-3'
	R: 5'-GGCATGTCACTCAGGTCCA-3'
Cyclin D1	F: 5'-TTCCTGCAATAGTGTCTCAGTTG-3'
	R: 5'-AAAGGGCTGCAGCTTTGTTA-3'
Beta-catenin	F: 5'-ACAGCACTCCATCGACCAG-3'
	R: 5'-GGTCTTCCGTCTCCGATCT-3'
